# Usefulness of Sacral Sublaminar Wire for Low Transverse Sacral Fractures: Two Cases' Report

**DOI:** 10.1155/2017/7396564

**Published:** 2017-10-04

**Authors:** Tatsuro Sasaji, Hideki Imaizumi, Taishi Murakami

**Affiliations:** Department of Orthopedic Surgery, Osaki Citizen Hospital, No. 3-8-1, Furukawa Honami, Osaki-shi, Miyagi 989-6183, Japan

## Abstract

Low transverse sacral fractures are rare, with only two published reports regarding their surgery. The complication associated with surgery for sacral fractures is the prominence of implants. In addition, screw fixation below S3 is impractical. We performed posterior sacral fixation using S2 alar iliac (S2AI) screws and sacral sublaminar wires for low transverse sacral fractures. Case 1 was 65-year-old male with an S2-3 transverse sacral fracture. We performed laminectomy (S2-3) and passed ultrahigh molecular weight polyethylene (UHMWPE) cables from laminectomy area to the third posterior sacral foramina. We inserted S2AI screws and connected rods. We also tightened the UHMWPE cables. The implants did not protrude into skin. One year after surgery, the sacral fracture healed without any displacement. Case 2 was a 42-year-old female with an S2 transverse sacral fracture. We performed laminectomy (S1–3) and passed UHMWPE cables from laminectomy area to the third and fourth posterior sacral foramina. We inserted S1 pedicular screws and S2AI screws and connected rods. We also tightened UHMWPE cables. The implants did not protrude into skin. One year after surgery, the sacral fracture healed without any displacement. We consider sacral sublaminar wires to be useful bone anchors in lower sacrum.

## 1. Introduction

Transverse sacral fractures account for only 3%–5% of all sacral fractures, with the rest being longitudinal sacral fractures [1, 2]. High transverse sacral fractures occur more frequently than low transverse sacral fractures [2]. Low transverse sacral fractures are reportedly considered to be relatively rare.

Selecting a treatment for sacral fractures is primarily based on the fracture pattern and neurological status of the patient [3]. For high transverse sacral fractures, various techniques such as Harrington rod [4–7], Luque method [8], lumbopelvic fixation [9], and locking plate [10] have been developed. The lower part of the sacrum is not involved in the transmission of weight from the lower extremities to the spine, and low transverse sacral fractures are considered stable [2]. For low transverse sacral fracture, there have been only two reports regarding surgical repair, locking plate [10], pedicular screws [7], and hook systems [7]. In the report of the locking plate technique, screws are inserted into the sacral midline [10]. Midline sacral screws have a risk for impinging on sacral nerves. Pedicular screw and hook placement have a risk for protrusion into the sacral skin. Therefore, we selected an alternative surgical method for incapacitated patients.

The reported complication regarding surgery for sacral fractures is that prominent hardware may be a source of discomfort or a nidus for skin breakdown postoperatively [11, 12]. In addition, because screw fixation below S3 is impractical [11], surgical methods for low transverse sacral fractures have not yet been established. Here, we performed internal fixation using an S2 alar iliac (S2AI) screw and sacral sublaminar wire (Schwend's technique) [13] and reported regarding the treatment course. On the basis of our surgical results, we described the usefulness of sacral sublaminar wires for repairing low sacral fractures. To the best of our knowledge, this is the first study to describe the usefulness of sacral sublaminar wires as low sacral bone anchor.

## 2. Case Presentation

### 2.1. Case  1

A 65-year-old male with schizophrenia had urinary retention and gait disturbance owing to sacral pain. His height was 173.7 cm, weight was 49.7 kg, and body mass index was 16.5 kg/m^2^ (weight loss) [14]. He complained of sacral pain and urinary retention 2 weeks after slipping and falling to the floor. Performing neurological examination was impossible because of schizophrenia. The urinary tract was not damaged. Thus, a diagnosis of neurogenic bladder dysfunction was made.

**Figure 1:**
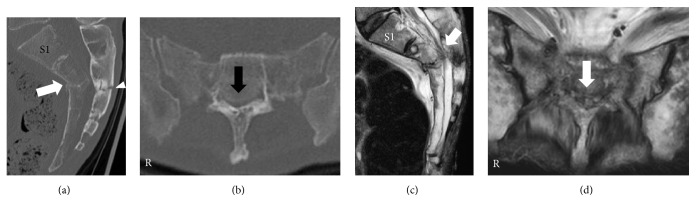
*Reconstructed computed tomography (CT) and magnetic resonance images of sacrum at first hospital presentation*. (a) Sagittal reconstructed CT image. (b) Axial CT image at S2-3 level. (c) Sagittal plane on a T2-weighted image. (d) Axial plane on a T2-weighted image. Fractures in the sacral vertebral body (white arrow) and lamina (white arrowhead) can be observed (a). Radiological findings were consistent with a Roy-Camille classification type I fracture [5]. Sacral canal was narrowed by the protruding sacral vertebral body (black arrow) (b). This radiological finding was diagnosed as a Denis Zone III sacral fracture [15]. Sagittal and axial plane on T2-weighted images showed that sacral nerves were compressed by the vertebral body (white arrow) (c, d).

**Figure 2:**
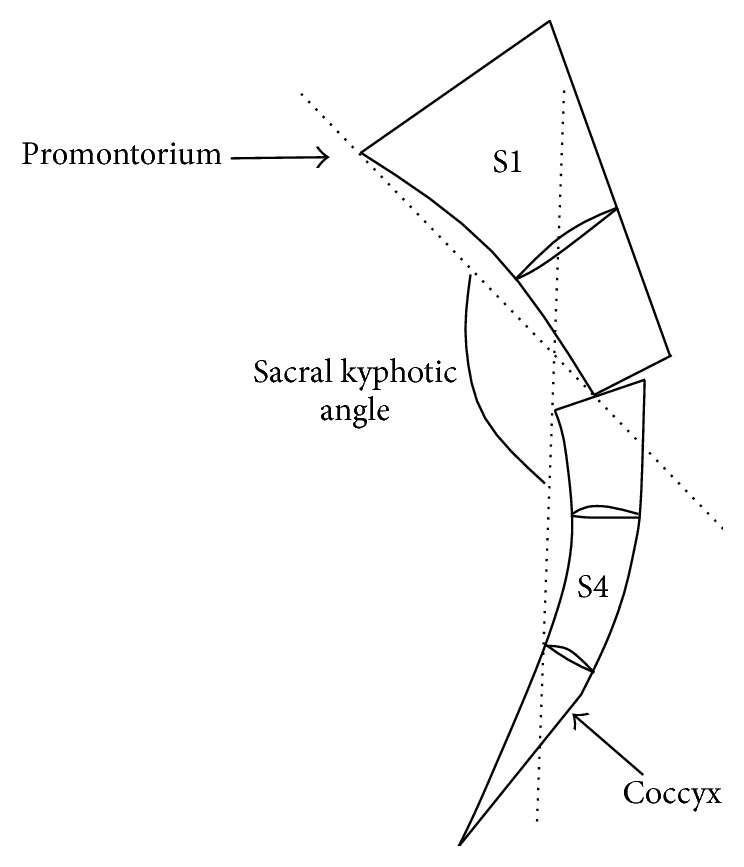
*Schemata of sacral kyphotic angle*. We defined the sacral kyphotic angle as the angle between the line from the sacral promontorium to the anterior edge of the proximal fracture fragment and the line from the anterior edge of the distal fracture fragment to the caudal edge of the fourth sacral body in a midline sagittal reconstructed computed tomography image.

Sagittal reconstructed computed tomography (CT) revealed that both sacral lamina and vertebral body were fractured and displaced into the sacral canal at the S2-3 level (Figure 1(a)). The sacral kyphotic angle was 110 degrees (Figure 2). Axial reconstructed CT revealed that sacral canal was narrowed by the protruding sacral vertebral body (Figure 1(b)). Sagittal and axial plane magnetic resonance imaging (MRI) revealed that sacral nerves were compressed by the angulated sacral vertebral body and lamina on T2-weighted images (Figures 1(c) and 1(d)). We determined that this fracture was unstable and would subsequently become a sacral kyphotic deformity. Our treatment plan was a decompression surgery with stabilization.

**Figure 3:**
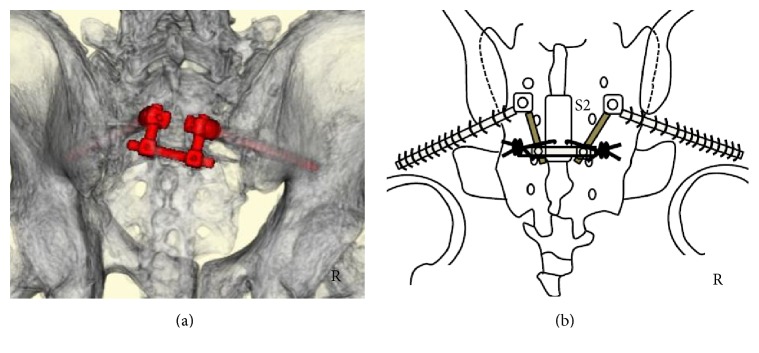
*Postoperative three-dimensional computed tomography (CT) and schemata*. (a) Three-dimensional CT. (b) Operation schema. Three-dimensional CT showing S2-3 laminectomy and S2AI screws and rods (red color) (a). Surgical method (b): we performed S2-3 laminectomy and then passed ultrahigh molecular weight polyethylene (UHMWPE) cables from the central laminectomy area to the third posterior sacral foramina. We inserted S2AI screws and connected the rods, after which the UHMWPE cables were tightened to the rods.

The second and third sacral laminae were explored through a straight posterior midline approach. Laminectomy of the second and third laminae was performed using a burr. No hematoma was observed. The sacral nerve roots were not disrupted. We passed ultrahigh molecular weight polyethylene (UHMWPE) cables (the NESPLON® Cable System, Alfresa Pharma Corporation, Osaka, Japan) from the central laminectomy area to the third posterior sacral foramina using a sublaminar wire technique. We inserted bilateral S2AI screws and connected the rods. Finally, we tightened the UHMWPE cables to the rods (Figures 3(a) and 3(b)).

**Figure 4:**
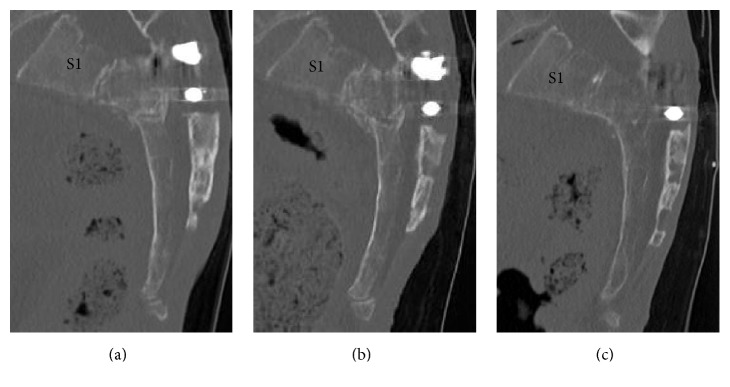
*Follow-up sagittal reconstructed computed tomography (CT) images of the sacrum*. (a) One-month sagittal reconstructed CT image. (b) Three-month sagittal reconstructed CT image. (c) One-year sagittal reconstructed CT image. The second-third sacral vertebral body exhibited a high-density area (a). The high-density area in the second-third sacral vertebral body increased (b). The second-third sacral vertebral body had united without any displacement (c).

One year after the surgery, the patient returned to normal daily life without a cane, although urinary retention persisted. One- and 3-month postoperative follow-up sagittal reconstructed CT revealed high-density areas in the second and third sacral vertebral bodies (Figures 4(a) and 4(b)). One-year postoperative follow-up sagittal reconstructed CT revealed that the sacral vertebral bodies had united without any displacement (Figure 4(c)). The sacral kyphotic angle was 108 degrees and the sacral kyphotic deformity did not deteriorate (Figure 2).

### 2.2. Case  2

A 42-year-old female with anxiety neurosis presented with urinary retention, perineal numbness, and gait disturbance owing to sacral pain. Her height was 160 cm, weight was 37.65 kg, and body mass index was 14.7 kg/m^2^ (weight loss) [14]. She complained of urinary retention 10 days after slipping and falling to the floor. Neurological examination revealed normal muscle power in her lower extremities, with decreased ankle jerk. Sensory disturbance was detected in the perineal region. Urinary retention was severe, with loss of bladder urgency. On the basis of the above findings, we diagnosed neurogenic bladder dysfunction.

**Figure 5:**
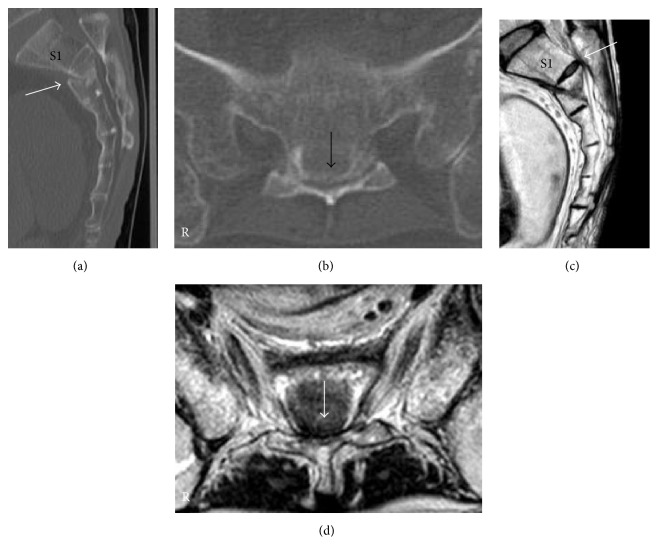
*Reconstructed computed tomography (CT) and magnetic resonance image of sacrum at first hospital presentation*. (a) Sagittal reconstructed CT image. (b) Axial CT image at S2 level. (c) Sagittal plane on a T2-weighted image. (d) Axial plane on a T2-weighted image. Displaced fractures in the second sacral vertebral body (white arrow) can be observed (a). This radiological finding was consistent with a Roy-Camille classification type II fracture [5]. Sacral canal was narrowed by the protruding sacral vertebral body (black arrow) (b). This radiological finding was diagnosed as a Denis Zone III sacral fracture [15]. Sagittal and axial planes on T2-weighted images showed that sacral nerves were compressed by the vertebral body (white arrow) (c, d).

Sagittal reconstructed CT revealed that the second sacral vertebral body was fractured and displaced into the sacral canal (Figure 5(a)). The sacral kyphotic angle was 107 degrees (Figure 2). Axial reconstructed CT confirmed that sacral canal was narrowed by the protruding sacral vertebral body (Figure 5(b)). Sagittal and axial plane MRI revealed that sacral nerves were compressed by the angulated sacral vertebral body and lamina on T2-weighted images (Figures 5(c) and 5(d)). We determined that the fracture was unstable and would subsequently become a sacral kyphotic deformity. Thus, we planned to perform decompression surgery with stabilization.

**Figure 6:**
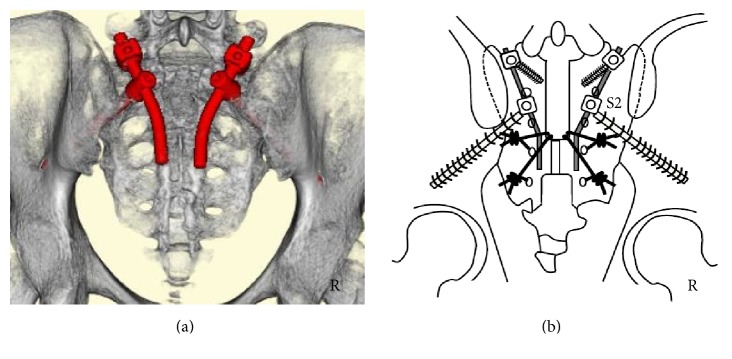
*Postoperative three-dimensional computed tomography (CT) and schema*. (a) Three-dimensional CT. (b) Operation schema. Three-dimensional CT showing S1–3 laminectomy, S1 pedicular screws, S2AI screws, and rods (red color) (a). Operation method (b): we performed S1–3 laminectomy. We then passed ultrahigh molecular weight polyethylene (UHMWPE) cables from the central laminectomy area to the third and fourth posterior sacral foramina. We inserted S1 pedicular screws and S2AI screws and connected the rods, following which the UHMWPE cables were tightened to the rods.

The first, second, and third sacral laminae were explored through a straight posterior midline approach. Laminectomy of the first, second, and third sacral laminae was performed using a burr. No hematoma was observed. The sacral nerve roots were not disrupted. We passed UHMWPE cables from the central laminectomy area to the third and fourth posterior sacral foramina using a sublaminar wire technique. We inserted bilateral S1 pedicular and S2AI screws and connected the rods. Finally, we tightened the UHMWPE cables to the rods (Figures 6(a) and 6(b)).

**Figure 7:**
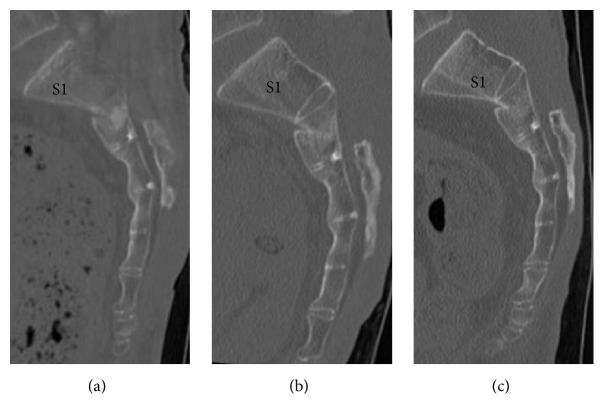
*Follow-up sagittal reconstructed computed tomography (CT) images of the sacrum*. (a) One-month sagittal reconstructed CT image. (b) Three-month sagittal reconstructed CT image. (c) One-year sagittal reconstructed CT image. The second sacral vertebral body was not displaced (a). The second sacral vertebral body contained a high-density area (b). The second sacral vertebral body had united without any displacement (c).

The patient's perineal numbness improved immediately after surgery. One month after surgery, she was able to urinate, and, 3 months after surgery, her bladder function had completely recovered. Three months after surgery, implant infection occurred. We considered that the fracture became stabilized at that time. Thus, we removed all implants. Following the surgery, the infection was healed. One year after the surgery, she returned to normal daily life without a cane. One- and three-month postoperative follow-up sagittal reconstructed CT revealed a high-density area in the second sacral vertebral body (Figures 7(a) and 7(b)). One-year postoperative follow-up sagittal reconstructed CT revealed that the second sacral vertebral body had united without any displacement (Figure 7(c)). The sacral kyphotic angle was 105 degrees and the sacral kyphotic deformity did not deteriorate (Figure 2).

## 3. Discussion

We believed that lumbopelvic fixation was not indicated in low sacral fractures because such a fixation would not be effective in these cases. Moreover, there was no vertical instability in these cases. We decided to perform internal fixation across the fracture line. These patients were severely incapacitated: therefore, a low-profile sacral bone anchor was essential. We selected S2AI screws as the cranial bone anchor and sacral sublaminar wire as the caudal bone anchor.

The S2AI screw is a sacropelvic instrument that provides a secure distal foundation [16]. The S2AI screw is a low-profile implant that minimizes implant prominence and tissue dissection [17–19]. Previous reports have determined that the S2AI screw was a strong and low-profile sacral bone anchor. Before operation, we were afraid that our patients would not follow our postoperative program and we needed a strong bone anchor to withstand their free behaviors. Thus, we selected the S2AI screw as a cranial bone anchor, resulting in good surgical outcomes.

The high-risk sacral midline locking screw [10] and protruded pedicular screw and hook system [7] were rejected as low sacral bone anchors. We also required another low sacral bone anchor. Schwend et al. reported the use of a sacral sublaminar wire for lumbar spondylolisthesis surgery [13]. On the basis of previous successful surgical outcomes, we selected a sacral sublaminar wire as caudal sacral bone anchor. In these incapacitated cases, the sacral sublaminar wire did not protrude into the skin and maintained a sacral kyphotic angle. Based on the successful results, we consider that a sacral sublaminar wire will overcome any reported postoperative problems to act as a useful bone anchor of low sacrum.

## 4. Conclusion

For low transverse sacral fractures, sacral sublaminar wire was low profile and could maintain the sacral alignment. Sacral sublaminar wire can be a useful low sacral bone anchor.
